# Traditional knowledge among Zapotecs of Sierra Madre Del Sur, Oaxaca. Does it represent a base for plant resources management and conservation?

**DOI:** 10.1186/1746-4269-8-24

**Published:** 2012-07-12

**Authors:** Azucena de Lourdes Luna-José, Beatriz Rendón Aguilar

**Affiliations:** 1 Instituto de Recursos Naturales, Colegio de Postgraduados, Km. 36.5 Carretera México-Texcoco, Montecillo, Texcoco, Edo. de México, 56230, Mexico; 2 Departamento de Biología, Universidad Autónoma Metropolitana, Avenida San Rafael Atlixco # 186, Col, Vicentina, C.P. 09340, México, D.F., Mexico

**Keywords:** Traditional ecological knowledge, Ethnoclassification, Zapotecs, San Agustín Loxicha, Ethnobotany

## Abstract

Traditional classification systems represent cognitive processes of human cultures in the world. It synthesizes specific conceptions of nature, as well as cumulative learning, beliefs and customs that are part of a particular human community or society. Traditional knowledge has been analyzed from different viewpoints, one of which corresponds to the analysis of ethnoclassifications. In this work, a brief analysis of the botanical traditional knowledge among Zapotecs of the municipality of San Agustin Loxicha, Oaxaca was conducted. The purposes of this study were: a) to analyze the traditional ecological knowledge of local plant resources through the folk classification of both landscapes and plants and b) to determine the role that this knowledge has played in plant resource management and conservation. The study was developed in five communities of San Agustín Loxicha. From field trips, plant specimens were collected and showed to local people in order to get the Spanish or Zapotec names; through interviews with local people, we obtained names and identified classification categories of plants, vegetation units, and soil types. We found a logic structure in Zapotec plant names, based on linguistic terms, as well as morphological and ecological caracteristics. We followed the classification principles proposed by Berlin [6] in order to build a hierarchical structure of life forms, names and other characteristics mentioned by people. We recorded 757 plant names. Most of them (67%) have an equivalent Zapotec name and the remaining 33% had mixed names with Zapotec and Spanish terms. Plants were categorized as native plants, plants introduced in pre-Hispanic times, or plants introduced later. All of them are grouped in a hierarchical classification, which include life form, generic, specific, and varietal categories. Monotypic and polytypic names are used to further classify plants. This holistic classification system plays an important role for local people in many aspects: it helps to organize and make sense of the diversity, to understand the interrelation among plants–soil–vegetation and to classify their physical space since they relate plants with a particular vegetation unit and a kind of soil. The locals also make a rational use of these elements, because they know which crops can grow in any vegetation unit, or which places are indicated to recollect plants. These aspects are interconnected and could be fundamental for a rational use and management of plant resources.

## Background

Traditional knowledge of indigenous or local people involves specific perceptions, beliefs and customs about natural environments [1,2]. Traditional knowledge is constructed by the close interaction between people and their environment through the daily use and management of natural resources and productive processes. It supports the subsistence activities of those groups that directly depend on the resource base [3,4]. Knowledge comprises the ways in which people categorize, code, process and impute meaning to their experience. These processes are based on an existing conceptual framework and are affected by the skills, interests, experiences, preferences, resources and patterns of social interactions that are characteristic of any particular group of individuals [5]. In this sense, traditional knowledge is the result of complex processes concerning social, cultural, institutional and ecological factors.

Traditional systems of classification or ethnoclassifications are expressions of traditional knowledge [6,7]. Knowledge generated through daily life interactions with plants and animals, as well as physical elements like climate and soil, is structured and integrated in different ways [8-10]. Many authors have analyzed the nature of these folk systems in terms of how they are structured [11]. Whereas Berlin and his collaborators argue that these systems follow a hierarchic and inclusive system, based mainly on distinctiveness or salience, for others, utilitarian features are the base of them; even more, some argue that in certain traditional cultures there are only proto classifications [8,9,12,13].

In Mexico, traditional folk systems of classification have been analyzed since the 1970’s, but few of them have been published. Some outstanding studies are those on ethnomycology among the Purépecha of Michoacán [14]; traditional nomenclature of plants, animals, soil, climate among Huaves of Oaxaca [15]; ecological ethnoclassification of the Chinantec and Mixe people of Oaxaca [16]; botanical nomenclature and structure of plant and animal classification of the Totonacos of Veracruz [5]; traditional classification and nomenclature of columnar and globular cacti among the Mixtec of Oaxaca [17], and a general analysis of classification in Maya culture [18]. The absence of these kinds of studies is paradoxical due to the high diversity of ethnic groups in Mexico that preserve local and ancestral traditions and customs.

### Zapotec ethnoclassification

Zapotecs live in four geographic areas of Oaxaca, the Central Valley, the Itsmo, the Northern Sierra and the Southern Sierra [19]. Although the Zapotec represent the third most numerous ethnic group in Mexico, including 8% of the national ethnic speakers in the nation [19-21] and are one of the most important cultures within Mesoamerican civilizations [22], there are few studies analyzing Zapotec traditional classification systems.

In the middle of the XVI century, Fray Juan de Córdova described and analyzed the names of animals and plants used by Zapotecs in the Valley of Oaxaca [23]. According to him, Zapotecs classify animals based on locomotion (e.g., walk, fly), habitat, size, behavior and their similarities with other organisms. For example, **máni péche** include ferocious animals (jaguar, ocelote), while máni péla refer to animals without legs (snakes, worms). The animal names in Zapotec began with the word **pe(be) o pi(bi)**, to indicate that they had a vital force that lead them to move. Plant classification was based on their usefulness, flavor and similarities with other plants. Messer [24] described the traditional plant classification among the Zapotec of Mitla, in the Central Valleys. She recorded five life forms, one of them corresponding to maize. Messer also showed how Zapotec terms refer to the plant growth stages and soil types where they grow. Brown and Chase [25] focused on Zapotec animal classification, among the people of Juchitán. In a similar way, the Zapotec of Santiago Xanica [26], identified and distinguished differences between mammals, birds and insects. Hunn and Acuca [27] developed a comparative analysis of Zapotec vocabulary used in present-day San Juan Mixtepec versus that reported in XVI century by Fray Juan de Córdova. Hunn later analyzed other aspects of Zapotec classification in the same locality [22]. While the modern Zapotec classification system includes 653 plant categories at a terminal level, more than 310 animals and 38 fungi, Córdova documented 309 plants, 290 animals, and 8 fungi. There are many patterns shared by both classifications, a comparision at life-form level shows these similarities (Table 1). 

**Table 1:** Comparison of Life-forms recognized among five Zapotec communities of Oaxaca

**Biological form**	**Trinidad Buenavista**	**Santiago Xanica**^**a1**^	**San Juan Mixtepec**^**2**^	**Mitla**^**3**^	**Valles Centrales**^**4**^
Trees and shrubs	Ya’a	Yak	Yàg	Yahg	Yága
Climbs and lianas	Lús		IbÈ	Behúk	
Herbs	La’a bixhs	Quiish	Guizh	K^w^an	Quijxi
Grasses (zacate)	Ixhs			Gishi	
Magueyes	Dob,Yes	Toob	Dòb		Toba
Canegrasses	Yií	Shiil			
Palms	Yiin				
Quelites	Yed *				
Bromeliads	Bla,bla lo ya				
Orchids	Goo lad ya, Xhil				
Ferns	Yoóh				
Mosses, hepatics, lichens	Mbaxhs				
Flowers	Iyé *	Kiée	Guièe		Guije
Fruits		Nguith shlea			
Mushrooms		Meí			
Nopales		Blaa			
Tuberous roots and corms		Kú			Còo
Leaves			Blâg		
Medicinal herbs			Ncuàan		Nocuana
Beans			Bziàa		
Corns				Yähl	

*Terms applied to groups of plants that have utilitarian value in the communities of San Agustín Loxicha, while in other communities of Oaxaca are reported as life-forms.

^a^Names applied to each plant form, but it is not indicated like this by authors.

^1^Cruz and Cruz, 1992; ^2^Hunn and Acuta, 2001; ^3^Messer, 1978; ^4^Marcus and Flannery, 2001.

The present study was conducted in the Zapotec municipality of San Agustín Loxicha, Pochutla district, in the Sierra Sur of Oaxaca, within the 129 Terrestrial Priority Region of Mexico, [28]. Zapotecs of this region depend on agriculture and gathering of forest products for their subsistence. In consequence, their interaction with nature in daily life has allowed the construction of knowledge about plant resources and a relative distinction of nature’s discontinuities, which are reflected in the existence of a traditional plant classification, as indicated in previous studies in this region [29-36]. The purposes of this study were a) to describe the traditional ecological knowledge of local resources through the folk classification of landscape and plants and b) to determine the role that this knowledge has played in plant resource management and conservation.

## Methods

### Study site

**Figure 1:**
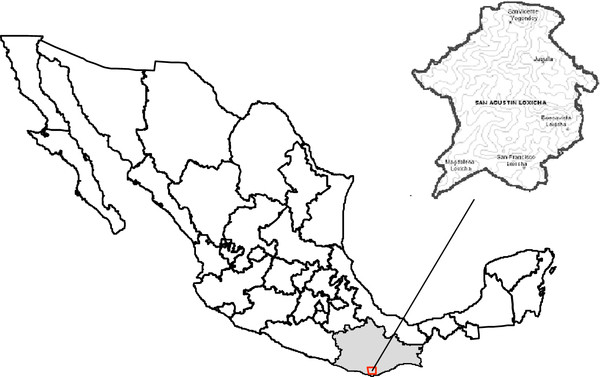
**Location of the San Agustín Loxicha municipality.** (Elaborated by: Gilberto Hernández C.). In this map, the location of the state of Oaxaca and the study area within it, is indicated.

The municipality of San Agustin Loxicha (Figure 1), is located at the SW of Oaxaca city (16°01^′^55^″^ N and 096°37^′^01^″^ W), in one of the troubled branches of the Sierra Madre del Sur. It occupies a total area of 389.1 km^2^. The administrative centre of the municipality is located at an altitude of 1,820 ma.s.l., though sparse settlements are found at lower and higher altitudes. The distance between San Agustin Loxicha and Oaxaca City is 180 km [37] Average temperature in San Agustin Loxicha is 16°C, with a minimum of −3°C and a maximum of 2°C throughout the year at different elevations [27]. Annual rainfall corresponds to 1,500 mm [38]. Soils are typically Regosol eutric and Cambisol distric, ortic and cromic with a fine texture. Rocks are metamorphic and date from the Precambrian period [39]. Vegetation varies in an altitudinal gradient from 330 to 2,250 masl [28,37], and seven vegetation units [31] are found: pine forest, pine–oak forest, oak forest, cloud forest, evergreen forest, tropical subdeciduous forest, and tropical deciduous forest [29-32]. 

Recent research indicates that Zapotecs in this region were established four centruries ago by people from Miahuatlan, specifically from San Agustin Mixtepec, who moved to the south [40]. Even when they exhibit a positive population growth rate, from the last three decades to present, a high rate of emigration (mostly seasonal migration) has been observed, with the consequential abandonment of their lands.

In 2005 (INEGI 2005), the municipality of San Agustin Loxicha had 17,823 people living in 8 municipal agencies and 61 colonies or barrios. Only 6% of the people live in two of the most important communities: San Agustín Loxicha and Tierra Blanca. Almost 80% (14125) of the people speak Zapotec, and 77% of them are bilingual, while the rest are only Zapotec speakers.

At higher elevations, the main activity is coffee cultivation in traditional systems in which coffee is intercropped with other plants like avocado (*Persea americana* Mill) (Lauraceae), guava (*Psidium guajava* L.) (Myrtaceae) and mango (*Mangifera indica* L.) (Anacardiaceae) under the cover of native tree species. Maize cultivation is also practiced in a traditional system, managing different native varieties of maize (*Zea mays* L.) (Poaceae), bean (*Phaseolus vulgaris* L.) (Fabaceae), and squashes (*Cucurbita* spp.) (Cucurbitaceae); farmers also promote or tolerate other edible non-crop species like chepil (*Crotalaria longirostrata* Hook & Arn.) (Fabaceae) and hierbamora (*Solanum nigrum* L.) (Solanaceae) within their maize fields. At lower elevations coffee is intercropped with guanabana (*Annona muricata* L.) (Annonaceae), several citrus species (*Citrus* spp.) (Rutaceae) and sugarcane (*Saccharum officinarum* L.) (Poaceae). Finally, at the lowest altitude, farmers cultivate maize in the traditional milpa system intercropping it with commercial species like Jamaica Roselle (*Hibiscus sabdariffa* L.) (Malvaceae) and watermelon (*Cucumis melo* L.) (Cucurbitaceae) in addition to various edible herbs [33,34,41]. Forest management is another important activity because different products are obtained for local or regional consumption [36].

The municipality of San Agustin Loxicha is located within the 129 Terrestrial Priority Region of Mexico, named Sierra Sur y Costa de Oaxaca, which is defined as a highly preserved area in terms of plant cover [28]. It is an area unexplored in terms of ethnobiological and ecological studies, until the last ten years, when few studies have been developed [29-36].

### Fieldwork

#### Selection of communities

Fieldwork was conducted during 2004 and 2005 in five communities of San Agustin Loxicha: Juquilita, Magdalena Loxicha, San Francisco Loxicha, Trinidad Buenavista, and San Vicente Yogondoy (Figure 1; Table 2). To select the communities where fieldwork would be conducted, we considered two criteria determined in a previous study [29,35]: that communities were distributed throughout the whole altitudinal interval in order to represent the floristic diversity present in the entire municipality, and communities had a minimum of 300 inhabitants. Data from the population census was used to select these communities [42]. The project was presented to the local authorities of the selected communities in order to ask their permission, and to facilitate the execution of the research. They were completely accorded with our presence. In some cases, they called the people of the community to an assembly in order to introduce us to them. Part of the successful welcome to the communities was due to the fact that the main author is a member of one of the communities and speaks Zapotec. In the communities of San Francisco Loxicha and Juquilita the local authorities faciliated household visits in order to conduct the interviews. 

**Table 2:** Geographic location, physical and economic characteristics of the five communities studied in the municipality of San Agustín Loxcicha, Sierra Madre del Sur, Oaxaca (PF, pine forest; POF, pine-oak forest; OF, oak forest; SDF, subdeciduous forest; CMF, cloudy mountain forest; EGF, evergreen forest; DF, deciduous forest)

**Community**	**Climate**	**Soil unit**	**Geographic coordenates**	**Altitude (m.s.n.m)**	**Vegetation units**	**Crops**	**Number of inhabitants**
Buenavista Loxicha	C(w2)(w)A(C)m(w)A(C)w2(w)Aw2(w)	LitosolCambisol eútricoCambisol crómicoCambisol húmicoLuvisol crómicoFeozem háplicoFeozem lúdicoAcrisol húmico	N 15°01^′^55^″^	1450	PF, OF, POF. CMF, EGF, SDF	coffee	2495
			W 96° 37^′^01^″^				
Juquilita			N 16° 01^′^ 24^″^	2050	POF	Maize in association with squash and different varieties of beans	356
			W 96° 34^′^ 53^″^				
San Francisco Loxicha			N 15°54^′^15^″^	480	SDF, CMF	Coffee,maize in association with Jamaica roselle, different varieties of bean	1723
			W 96° 36^′^10^″^				
Magdalena Loxicha			N 15°53^′^55^″^	330	DF	Maize in association with Jamaica roselle, different varieties of bean	2464
			W 96° 41^′^28^″^				
San Vicente Yogondoy			N 15°53^′^55^″^	1460	PF, OF	Maize in association with Jamaica roselle, different varieties of bean	922
			W 96° 41^′^28^″^				

#### Interviews

We selected 20 people from each community, based on the facility they offered to the interview. We maintained the same proportion of men and women. Since according to Berlin [12] six year old children recognize generic plant elements, we included in our study the perception of all members of the community, selecting people from 8 to 80 years old [43].

We contacted people in their houses, in the streets, or in the small markets, and explained to them the objectives of our research and told them that the interview lasted between 20 and 30 minutes. In most cases, people agreed to participate. In many cases, we conducted the interviews on casual walks to the milpa, or the coffee plantations, and collected plants with them. When we had doubts about a plant identity, we asked them to collect it, in order to determine if this species was different but had the same name.

The questionnaire consisted of four questions: what are the Zapotec and/or Spanish names of the plants that are gathered or cultivated; what are the uses of each plant mentioned; what is the recognized life form; and where and how are the habitats where the plant species occurs. During the interview, we asked people to describe plants, in order to know what species they were talking about. It is important to indicate that previous plant vouchers had been collected, as well as an existing list of local names (Zapotec and/or Spanish), from field studies developed in this and two other municipalities of this region during 2002–2004 (Candelaria Loxicha and Pluma Hidalgo). Most of them corresponded to floristic and structure characterization of different plant communities (pine–oak–subdecidous forest association, subdeciduous forest, cloudy forest). The others consisted of a characterization of floristic composition of traditional coffee systems, and a study of forest resources [30-33,36]. In all cases, Zapotec or Spanish names of plant species were recorder and voucher specimens were collected. In this study, we verified syntaxys, including the prefix used for each life form. In those cases where prefix was not incorporated, or plant species only had a Spanish name, we asked people to identify which life form that species belonged to. The zapotec names were reviewed by an elementary school teacher.

We also asked them about what characteristics they use to classify soil and vegetation types. In the case of soil, only color and texture were considered for classification. In the case of vegetation types, they considered plant cover, tree size, plant species, altitude, and soil characteristics.

With the information gathered from the interviewers, we followed Berlin’s taxa system, based specifically on plant names, as well as morphological and ecological attributes. The linguistic component was a factor in grouping plant elements. We tried to establish nomenclatural relationships, taking into account most of the aspects discussed by Berlin, like mono or polytypic, primary, secondary, productive or unproductive names [12]. For generic taxa, we considered the lexical terms applied to each plant species. We also verified if people grouped them in the same term, asking them “if plants are sisters or if they are almost the same”, this concept was frequently used by the locals themselves. This identifying process was also used with specific/varietal names: we “elaborate” with them the classification of those species from the life form to the last category they considered. In the case of polytypic names, we included them in those cases when one Zapotec name was applied to different Latin species. In the case of productive names, we considered them when Zapotec terms describe in an explicit way some plant attributes whereas unproductive names correspond to Zapotec terms that do not correspond with any of them.

Since plant names, as well as the logic followed to group them, were documented in previous studies to the present [30-33,36] we found that this classification was consistent and represented the cognitive processes that people have organized through generations [11].

Voucher specimens were collected in the study area during field trips and deposited in the herbariums of Universidad Autónoma Metropolitana (Ramón Riva y Nava –Esparza) and the Universidad Nacional Autónoma de México (MEXU).

## Results

### Landscape classification

**Figure 2:**
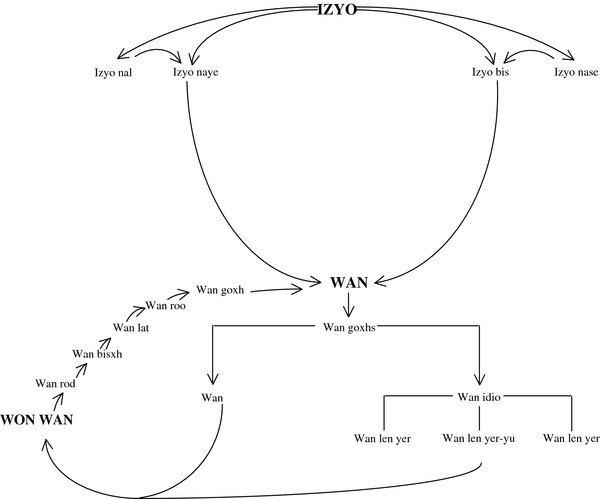
**Relationship among different vegetation units, based on physical factors, plant composition, and disturbance degree.** This figure indicates the way Zapotecs of San Agustín Loxicha perceive their environment. Zapotecs use the term (**wán**) to vegetation, but they also use concepts like **izyo bixhs**’, **izyo nayee**, which means dry earth and green earth, respectively. They also recognize that vegetation distribution varies depending on temperature and humidity, and these differences are indicated with concepts like **izyo nasú** o **nacee** or **izyo nal** for plants that grow in dry and hot places, and others in cold and wet places, respectively. The term **wán** designs all plant components and based on physiognomy and disturbance level, they are grouped in three Zapotec categories: **wán nosa**, includes cloudy mountain, evergreen, subdeciduous and deciduous forest, characterized by a complex mixture of plants. In contrast, vegetation conformed by template elements is named **wán idio**, which comprises the next vegetation units: **wán len yer**, which corresponds to pine forest, where *P douglasiana,P. maximinoi* y *P. oocarpa* are the dominant species; oak forest, **wán len you** or **wán ya you** where *Quercus candicans, Q. crassifolia. Q. elliptica, Q. nixoniana, Q. obtusata, Q. ocoteifolia Q. peduncularis, Q. polymorpha, Q. subspathulata* y *Q. uxoris*; spp. are the dominant elements, and **wán len yer** – **you**, where *Pinus* and *Quercus* are codominant species. The term **Won wán** is applied to secondary vegetation, derived from **wán gosh** that has been subject to human manipulation and then left to rest. Different stages can be recognized: **wán rod**, corresponds to small shoots; **wán bixhs** or **wán lu**, small herbs; **wán lat**, small shrubs and trees; **wán goxh,** tall and thick trees, which is also applied to natural vegetation areas. If lianas with spines grow in the area, they use the term **wán do’o**. If plant cover has been modified by introduction of coffee plants, it is named **wán lo ya’a café**.

Zapotecs use the term **isyo** for naming general environmental units; for instance, they use the term **isyo bixhs’** for referring to dry lands and **isyo nayee** for referring to relatively more humid land with green plant cover. They recognize that temperature and humidity influence plant distribution and that there are some plants growing exclusively in hot areas (**isyo nasú** or **isyo nacee**) and others growing in colder and wetter environments (**isyo nal**). Zapotecs also use the term **wán** for naming all forest or vegetation types, but use particular epithets for referring to specific types (Figure 2).

**Wán** applies to plant components and based on physiognomy and disturbance level, they are further grouped into three Zapotec categories: **wán nosa** (mixed vegetation), includes cloudy mountain, evergreen, subdeciduous and deciduous forest, it’s characterized by a complex mixture of plants. The Zapotec do not use a specific term for each of these vegetation units. Vegetation conformed by temperate elements is named **wán idio** (mountain vegetation) and includes the following vegetation: **wán len yer** (pine forest), where *P douglasiana* Martínez*, P. maximinoi* H.E. Moore y *P. oocarpa* Schiede & Schltdl. are the dominant species; **wán len you** or **wán ya you** (oak forest), where *Quercus candicans* Née*, Q. crassifolia* Humb. & Bonpl.*, Q. elliptica* Née*, Q. nixoniana* S. Valencia & Lozada-Pérez, *Q. obtusata* Bonpl.*, Q. ocoteifolia* A. Camus, *Q. peduncularis* Née*, Q. polymorpha* A. DC.*, Q. subspathulata* Trel., and *Q. uxoris* McVaugh are the dominant elements, and **wán len yer**–**you** (pine-oak forest), where *Pinus* and *Quercus* are codominant species.

The term **won wán** is applied to secondary vegetation derived from **wán gosh,** that has been removed, and then left to rest. Different stages can be recognized: **wán rod**, corresponds to small shoots; **wán bis** or **wán lud** (small vegetation), small herbs; **wán lat** (thin vegetation), small shrubs and trees; **wán goxh** (old vegetation), tall and thick trees, which is also applied to natural vegetation areas. If lianas with spines grow in the area, they use the term **wán do’o**. If plant cover has been modified by introduction of coffee plants, it is named **wán lo ya’a café**.

Zapotecs interviewed said that soil type also influences the landscape form. In general, soils present in the community are diverse. According to literature and soil maps [44,45], there are three main soil types: luvisol, regosol and dystric, crhomic and orthic cambisol. Traditional classification is based on texture and color. **Yü nagat (black soil)** corresponds to a fertile soil with a large accumulation of organic material where vegetation is abundant and/or productive and can be generally found in the pine forest. **Yü nguin** (sticky soil) is found mostly in the jungle, it is mixed with pine leaves and used to build adobe homes and braceros (to boil food). The sands around the rivers are known as **yü yuxhs** (sandy soil), and the white soils found in the lower regions are called **yü nequis** (white soil). **Queda** (rock) is a rocky soil with poor plant cover. Soil classification is also related to the crops that grow better on them. Thus, maize grows better in black, white and rocky soil, while coffee plantations are located mainly in clayish soils with high accumulation of organic matter (Table 3). 

**Table 3:** Soil classification and its relation to climate, vegetation and human activities developed on them

**Soil**	**Climate**	**Vegetation**	**Crop**	**Another activities**
Yü nagat (black soil, humus)	Izyo nal (template)	wán len yer (Pine forest), Wán len yer-yuo (Pine-oak forest), Wán len yuo(Oak forest)	Maize with squash and beans	Forest wood
Yü ngüin (clayish soil)		wán (Cloud mountain forest, evergreen forest y subdeciduous forest)	Coffee	Coffee system
Yü yuxhs (Sandy soil with small rocks), Yu nequis (White soil)	Izyo nase (template/ tropical, humid)	wán (Cloud mountain forest, and subdeciduous forest)	Coffee, maize with squash and beans	
Yü nequis (White soil), Queda (rocky soil, without plant cover)	Izyo bixhs (hot, dry)	wán (deciduous dry forest)	Maize in association with Jamaica roselle, and Sollamiche palms	Livestock

### Ethnobotanical system of classification

We recorded 757 plant names. Most of them (67%) have an equivalent Zapotec name and the remaining 33% had mixed names with Zapotec and Spanish terms. Plant species named and classified by Zapotecs are mainly native, but some of them are species introduced to the region in pre-Hispanic or later times but play a basic role within the culture because they are part of their daily diet.

Plants are named and grouped in a hierarchic system based on ecological and morphological characteristics. People recognize plants from the tropics, the temperate areas, or those that are found in specific sites (e.g., pines and oaks are mainly distributed in temperate areas, so their name is related with their ecological distribution: **wán len yer** is pine forest and **wán len you** is oak forest).

**Figure 3:**
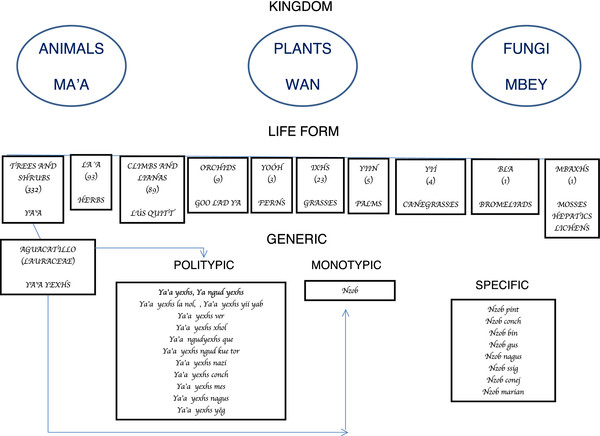
**In this context, the semantic structure of terms used for the 757 plants exhibit a regular pattern highly related to morphological, anatomical, physiological and ecological attributes, which allows us to group plants in a hierarchical structure.** Zapotecs clearly differentiate many fungi, identified as **mbey**, from animals, grouped under **ma’a**, and from plants, known as **wán** (vegetation). This term is also applied to the vegetation, which means that unlike individual beings, the plants are perceived as a whole and integrated into different systems (e.g., primary or secondary vegetation).

To classify plants in the different life forms, Zapotecs include morphological and physiological characteristics as well as anatomic structures or tastes. They differentiate tubers **(goo)** from roots **(loxhs),** or colorful petals (**iyé)**, from inflorescences like spadixes and spikes (**dob)**. Other structures are stems (**tronc)**, branches **(cosdxod)**, barks (**Xxäb)**, leaves (**Lla’a)**, fruits (**ngud)**, and seeds **(mbis)**. For example, many epiphytes have the prefix **goo** (e.g., **goo biuxhs** (*Bomarea edulis* (Tussac) Herb) (Astroemeriaceae); **goo xhil** (*Dioscorea mexicana* Scheidw.) (Dioscoreaceae), which make reference to the presence of tuber; in the case of agaves, the term **dob** makes reference to the large inflorescence (e.g., **ya’a dob bhied** (*Agave* spp.) (Agavaceae). In another example, plants with underground stems (like some cycads) are classified in the same way as orchids and dioscorids, or other plants that have tuber. **Goo ya’a** corresponds to *Manihot sculenta* Crantz (Euphorbiaceae), **ya’a goo xhil** to *Ceratozamia* aff. *longifolia* Miq. (Zamiaceae), and **goo malang** to *Colocasia esculenta* (L.) Schoot (Araceae), while **loxhs** corresponds to the rest of the plants that exhibit a normal root system (Figure 3).

In this context, the semantic structure of terms used for the 757 plants exhibit a regular pattern highly related to morphological, anatomical, physiological and ecological attributes, which allows us to group plants in a hierarchical structure. Zapotecs clearly differentiate many fungi, identified as **mbey**, from animals, grouped under **ma’a**, and from plants, known as **wán** (vegetation). This term is also applied to the vegetation, which means that unlike individual beings, the plants are perceived as a whole and integrated into different systems (e.g., primary or secondary vegetation).

In the second level of this hierarchical classification, we found that people always group plants in one of the seven recognized life forms. In most of the cases, life form is an explicit term of the name applied to each species: **ya’a**, is applied to trees and shrubs. In the nomenclature, this corresponds to the first term of the name (e.g. **ya’a tii,***Guazuma ulmifolia* Lam. (Sterculiaceae)**,** and **ya’a treen,***Croton draco* Schltdl & Cham.) (Euphorbiaceae). In some cases, the term **ya’a** is replaced by another enhancing a particular characteristic. Nevertheless, the plant is recognized by the life form. For example, banana is considered as a **ya’a** but people name it as **bdó**, because the main attribute is the fruit. In other cases, it is possible that plants introduced in recent times, weren’t incorporated into the classification system. **Lús,** is a term used for climbs and lianas. Some plants are included in this group based on their appearance. *Guarea* sp. (**lús quitt)** (Meliaceae) is a decumbent shrub, but it is included in this group because its branches extend and the thin stems have the resistance and ease to bend like vines to create an arch. *Baccharis trinervis* Pers. (**lús cunic**) (Asteraceae) is a branch-like weed/herb whose branches on occasion extend in the form of a guide. **La’a bixhs** is used to name herbs, even though this term is also applied to the tree and shrub leaves. **Ggoo lad ya** is used to identify orchids because it means “camote that grows on the tree”. **Goo** can also be applied as a generic term to name the corms, bulbs and tuberous roots; **yoóh,** means ferns; **ixhs,** grasses; **yiin**, palms; **yií,** canegrasses; **dob,** agaves; **bla**, bromeliads; **mbaxhs,** mosses, hepatics and lichens. We found that people sometimes grouped colorful flowers and “quelites” in separate categories (**iyé** and **yed**, respectively). Nevertheless, all of them are grouped in some of the previous categories, mostly **ya’a, la’a bixhs and lús** (Figure 3).

In the next level, the particular name given to each species is indicated as a generic term. In the case of monotypic names, diverse combinations of terms were found: simple terms in Zapotec (11 species; i.e., **fdión**, **ngüti**, **nij**); simple terms with a light modification since the words are borrowed from Spanish, generally with the elimination of the last letter (13 species, i.e., **mirt**, **mostrnz**, **manzan**); species that completely maintain their Spanish name (111 species, i.e., **bambú**, **sávila**, **cedrón, eucalipto, hierba de pollo**); Zapotec prefix (generally corresponds to the life form) and Spanish suffix (53 species, i.e., **ixhs limún**, **lús granad**, **la’a cancer**, **ya’a mang**, **iyé San José**) and Zapotec prefix and suffix (201 species, i.e., **la’a gaxhs**, **la’a ya mbesh**, **ixhs mbad, lús quitt, ya’a ngud khin**, **ya’a ngüid**, **iyé mengo**, **yed ñia**) (Figure 3).

We obtained 377 polytypic names, which correspond to 88 groups. In most cases they are species of different families, although in the case of the members of Melastomataceae, Fagaceae, Mimosaceae and Lauraceae, the names corresponds to the same family, but different genus or species. An example of this generic polytypic is represented by species of the Lauraceae family. People include all species, corresponding to different genus, in the generic term **ya’a yexhs**. Nevertheless, the morphologic attributes used to include them are the form of the trunk and the leaves, as well as the characteristic odor of the leaves (Table 4).

**Table 4:** Generic polytipic taxa represented by different species of Lauraceae

**Zapotec name**	**Spanish name**	**Scientific name**
**Ya’a yexhs, ya ngud yexhs**	Aguacate	*Persea americana* Mill.
**Ya’a yexhs la nol, ya’a yexhs yii yab**		*Nectandra cuspidata* Nees & Mart.
**Ya’a yexhs ver**	Aguacatillo	*Ocotea atacta* Lorea-Hern.
**Ya’a yexhs xhol**	Aguacatillo, palo guatoso	*Persea* aff. *donnell-smithii* Mez
**Ya’a ngudyexhs que**	Aguacate piedra	*Persea nubigena* L.O. Williams
**Ya’a yexhs ngud kue tor**	Aguacate de toro	*Persea* sp.
**Ya’a yexhs nazi**	Aguacate oloroso	*Persea* sp.
**Ya’a yexhs conch**		*Persea* sp.
**Ya’a yexhs mes**		*Persea* sp.
**Ya’a yexhs nagus**		*Persea* sp.
**Ya’a yexhs yëg**		*Persea* sp.

In this case, the generic term is **a’a yexhs.**

We found 23 primary productive names, which are chosen according to some characteristic of the plant, as in the case of **la’a arla**, leaf (**la’a**) bitter (**arla**). The name implies that the leaf is bitter. The non-productive names do not have any relation with the plant, as in the case of the “ear of lion”, which morphologically does not have any similarity with this feature. Of these last cases only 15 (2%) were found.

In the case of some taxonomic groups that are not often used, i.e. mosses, lichens and hepatics, a generic category exists that is based on the growth habit. Mosses and hepatics that grow in the superficial part of the ground are called **mbaxs lad ble**. Lichens that hang off the branches of trees are labeled **mbaxs lo ya’a**, and those that grow on the branches of coffee plants are **mbaxs lo ya’a cafe**.

In some cases, Zapotecs group plants that show marked perceptual similarities among the members, but they belong to different orders, or even more, to different divisions. For example, palms are subdivided in three generic taxa, and cycads are included in one of these. Thus, arborescent palms (Arecaceae) are grouped in the taxon **ya’a gaá**. Shrubby palms are divided in another two taxa, **ya’a xhil** (*Chamaedorea* aff. *Elegans* Mart.), which also included the cycad *Ceratozamia* aff*. longifolia* Miq. (named as **ya’a xhil goo)**, the palm **ya’a yiín** (*Cryosophila nana* (Kunth) Blume ex Salomon), and the arborescent fern **ya’a yoóh xhil** (*Cyathea costaricensis* (Mett. ex Kuhn) Domin). In the case of **ya’a yiín**, the recognized subdivision within the shrubby palms is due to their present distribution in the lower tropical regions.

Another example is the generic **yeg** that means pumpkin, which includes species of two families: Cucurbitaceae and Asclepiadaceae. Pumpkins (*Cucurbita* spp.) are named as **yeg**, while yeto, an Asclepiadaceae of the genus *Gonolobus*, is named **yeg na**, possibly due to the fruit appearance and life-form.

At the varietal level, in all cases, Zapotecs group the varieties of a species, a process similar to the infraspecific occidental classification, but also species belonging to the same genus. We recorded 137 species belonging to 36 varietal taxa, among which chile, pumpkin, bean, corn, grasses, banana, and cuiles are the most representative. One example corresponds to maize, which different names correspond to different varieties (Table 5, Figure 3). Most of the species that are located in this specific/varietal category correspond to cultivated plants.

**Table 5:** Varietal taxa recognized for different native varieties of *Zea mays.* Generic term is **Nzob**

**Zapotec name**	**Native variety**
**Nzob pint**	Pinto
**Nzob conch**	Azul
**Nzob bín**	Tepezentle
**Nzob gus**	Amarillo
**Nzob nagus**	Tablita, normal
**Nzob sig**	Delgado
**Nzob conej**	Conejo
**Nzob marian**	Magallano

## Discussion

### Landscape and soil classifications

Plant communities’ classification is similar in some aspects to scientific science. In ecology, they are classified in terms of their physiognomy (e.g., dominant species), composition or geomorphic features. In the present study, we found that Zapotecs classify landscape using physiognomy, composition and habitat criteria (altitude and climate). This aspect has been reported previously for other ethnic groups [16,46-48]. The recognition of these aspects is important because Zapotecs use them to establish different uses of plant communities: to limit their community, to obtain different products (plants or animals), to have places to rest and enjoy, to preserve some areas as a communal property. Nevertheless, plant communities are strongly related to the concept of earth, which in turn relates to the concept of soil. The earth determines what kind of vegetation can grow and its characteristics. The terms **isyo nayee (**green earth), and **isyo bixhs’** (dry earth), correspond to the first level from which they recognize or determine which plant components can be found. From here, the next perceptual associations identify the kind of crops and plants of the natural vegetation that can grow on the land (black soil grows coffee; sandy soils grow palms; red soils grow pine and oak forests), and indicate a deep and ancient knowledge that has played an important role not only in the Zapotec understanding of plants adaptive significance, but also in the integration of these soil–vegetation perceptions into their daily activities [49,50].

### Ethnobotanical system of classification

The Zapotecs recognize, name and classify not only those plants that are useful for them in a utilitarian context (i.e. medicine, food, firewood), but also those plants that do not have an immediate use. These are also important because some ecological or biological characteristic, like ecological dominance or some biological particularity (e.g., plants that grow in specific places), show and contribute to the integration of diverse biological and physical elements in the natural environment. Names of these species were reported in other papers by the same authors (e.g., *Bursera simaruba* (L.) Sarg. (Burseraceae), *Hymenaea courbaril* L. (Fabaceae), *Myriocarpa longipes* Liebm.) (Urticaceae) [35].

Zapotecs exhibit a deep traditional ecological knowledge in the Sierra Sur of Oaxaca in the sense that a fine classification exists, and also shows how they perceive their environment. Vascular plants, which include pteridophytes, gymnosperms and angiosperms, were classified with Zapotec terms, including life form, generic and in many cases, specific/varietal taxa. This classification shows a high correspondence with scientific terms and thus demonstrates the cultural, ecological, and economical importance of these plant elements. In the case of non-vascular plants, which include mosses, hepatics and lichens, they are grouped in Zapotec terms that include Linnean orders, families and species, making a very diffuse hierarchy for these organisms. Nevertheless, we cannot conclude that these plant elements have less importance to Zapotec culture, but rather are perceived in a different way by them. A comparative analysis with other studies, showed evidence that a traditional term corresponding to kingdom category is applied to some or all the non vascular elements. For example, among the purépechas [14] and the totonacos, a term exists that distinguishes mushrooms in a separate group [5] (although in the last ethnic group, there is no term assigned to the plants). These same finds have been recognized in other ethnic groups of Cameroon [51] and Venezuela [49]. Nevertheless, among the Wola, of New Guinea, there is no evidence of a term assigned to any element of the vegetable kingdom [46]. Martínez [50], comments that in Mesoamerican groups, a concept of plant does not exist. We believe that the idea of plant is strongly linked with vegetation or “monte” as other studies indicate [23,26,52]. Thus, this concept conforms a plant–vegetation duality, so it is more complex that the existence of a singular plant model.

Life form is a fundamental element in the traditional classifications since it reflects the distinction made between forms based on the visible morphology and ecological adaptations. There are different interpretations about what a “life form” is and which are the attributes that characterize this highly inclusive botanical category [10]. Nevertheless, there is a general agreement that they are recognized in different cultures. Different studies developed in Zapotec communities report among five to eight life forms [12,19,20,23,26,27] (Table 1). In all the cases, including this study, trees and shrubs are grouped in one category, possibly due to the fact that it includes the idea of wood and firewood. This is a similar pattern reported by Hunn [10] and discussed by Caballero and Cortés [18] and clearly indicates that life-forms are strongly linked to other aspects of Zapotec cosmovision, in this case with daily life activities and necessities of the people. More discussion must be made to define clue characteristics that must represent a life form within the indigenous perception, as Hunn [10] indicates. We also must consider Brown and Chase’s appreciation [25] about the role that modernization has on traditional cultures, which in part, is reflected in an increasing number of life forms.

As Berlin [12] indicates, generic taxa are the most numerous. It is important to mention that the majority of the elements are monotypic, which shows that a high proportion of them correspond with scientific taxonomy. Previous studies in Zapotec communities did not analyze this category, so we do not have any point of comparison. Nevertheless, Mapes et al*.*[14], Aparicio y García [5], Caballero and Cortés [18], and López-Franco [21], reported a similar pattern and reinforced the observation that these taxa have high levels of correspondence with occidental taxonomy. At the same time, the low percentage of species with names borrowed from Spanish or Spanish names indicate that most of the plant elements have been used long time before the Spanish Conquest.

Finally, specific/varietal names correspond to frequently used species in the community. Such is the case of corn, beans, pumpkins, tomatoes and chiles, which denotes an intensive selection process under domestication. Other examples of this fine perception and classification have been reported with *Manihot sculenta*[38], and with many cacti species like *Opuntia pilifera* F.A.C. Weber [53], *Stenocereous stellatus* (Pfeifer) Riccob., *S. queretaroensis* (F.A.C. Weber) Buxb., *S. pruinosus* (Otto) F. Buxb., *Escontria chiotilla* (F.A.C. Weber) Rose, *Polaskia chichipe* (Gosselin) Backeb. and *P. chende* Gibson & Horak [54].

### The role of traditional knowledge in plant resource management and conservation

The site of study is located in one of the 125 Terrestrial Priority Regions proposed in 2000 by the National Comitée for Biodiversity Knowledge and Use (CONABIO). This region presents a wide plant cover, conformed by different types of vegetation, but an important presence of pine–oak forest, cloudy mountain forest, and tropical subdeciduous forest.

Human activities are comprised by a farming system that includes the management of different land use units like maize, fallow land, coffee forest gardens, home-gardens and forest. The system integrates both subsistence and commodity production. Home-gardens, maize and fallow land are mainly used to produce food for self consumption while coffee forest gardens are used to generate cash income [55]. Coffee is cultivated under the canopy of natural or managed forest. The rest of the farm activities involve reduction of plant cover.

In this context, a high percentage of plants integrated to the folk systems are part of at least one of the farm systems, and they play specific roles within them: to shadow coffee shrubs, as an organic fertilizer in coffee systems and in some “milpa” areas as food, or medicine. Most of them are wild, so people must know where the plants exist, their phenology, abundance, and other traits, in order to get these resources. In this process, people make comparisons between plants, organize and classify them, and develop a holistic concept of each plant within the context of its environment. Many plants are favored over others, but all of them are important. Even when some plants are dangerous because they are toxic for humans or animals, they are considered in this holistic view [35]. This understanding of nature allows for the continued protection of natural resources even when they are subject to daily use.

The present study reinforced the findings analyzed by Toledo et al. [2], that highly preserved area in Mexico, in terms of biodiversity, are occupied by ethnic groups that make a rational use of natural resources.

## Conclusions

In the context of Zapotec culture, this fine system of classification reflects the knowledge that the Zapotecs have inherited from their ancestors and the importance of knowing the existence and function of the elements that are part of their local environment. In the context of scientific science, this traditional ecological knowledge has contributed enormously to preserve plant cover of a wide area of this municipality, even when the communities have been established more than a century ago. This is a consequence of the different strategies that they have developed through generations: daily use of many plants for food, medicine, timber, etc.; the traditional management of their productive systems (milpa with policulture and coffee plantations with native plants). These strategies emerge from their necessity to rely on their ambient vegetation. Because they understand their situation better than foreign people, they try to make the best decision about how to manage a particular plant or a particular plant community. Through experimentation they decide where to sow a particular crop and try to avoid negative effects (soil erosion, bad crop mix). It’s important to mention that increasing foreign pressures with low coffee prices, or low production of maize, have forced them to make some equivocal decisions like the use of agrochemicals, that kill edible or medicine plants that grow in the milpa, as well as the introduction of hybrids of maize instead of the maintenance of native races. Nevertheless, these are individual, more than collective, decisions that until this moment have not affected their ancestral and cumulative knowledge.

## Competing interests

Both authors declare that they have no competing interests.
